# Case report: Electrical impedance tomography-guided ventilator weaning in an obese patient with severe pneumonia

**DOI:** 10.3389/fmed.2024.1505114

**Published:** 2025-01-06

**Authors:** Yuxia Liu, Rui Liu, Bin Li, Libi Cai, Jieqi Hou, Wei Zhao

**Affiliations:** Intensive Care Unit, Zhongshan City People's Hospital, Zhongshan City, China

**Keywords:** electrical impedance tomography, ventilator weaning, pneumonia, respiratory failure, case report

## Abstract

**Background:**

Electrical impedance tomography (EIT) evaluates lung function by providing continuous, real-time monitoring of regional lung ventilation distribution to guide the restoration of lung ventilation. Patients with obesity who are dependent on mechanical ventilation often struggle with weaning. This case report highlights the potential of EIT to guide the weaning of ventilator-dependent patients in an obese patient with severe pneumonia.

**Case summary:**

A 23-year-old male obese patient was admitted to Zhongshan People’s Hospital in November 2023 due to progressive dyspnea. He suffered from recurrent fever and worsening oxygenation and had a 20+ year history of syringomyelia. Combined with abnormal computed tomography imaging, the discovery of a viral infection in the bronchoalveolar lavage fluid, and next-generation sequencing results, he was diagnosed with severe pneumonia. In addition to conventional treatment, EIT was used to guide him through postural changes and ventilation recovery. EIT-informed prone positioning improved ventilation distribution and facilitated successful ventilator weaning on day 26. The patient was discharged after recovering spontaneous respiratory function.

**Conclusion:**

EIT may help ventilator-dependent obese patients achieve tailored targets to improve ventilatory outcomes.

## Introduction

Obesity is at epidemic levels in the majority of countries in the world, significantly impacting respiratory mechanics and predisposing individuals to atelectasis and prolonged mechanical ventilation (1). Unfortunately, authorities’ guidelines lack specific recommendations for weaning obese patients from ventilation (2). One of the greatest challenges in critically ill patients with obesity is the optimization of mechanical ventilation, and the controversy surrounding weaning revolves around the utilization of PEEP/CPAP during spontaneous breathing trials (SBTs). Severe pneumonia often leads to respiratory failure because lung damage caused by infection reduces the efficiency of oxygen exchange. Patients with respiratory failure are unable to maintain normal breathing on their own and often rely on mechanical ventilation to support respiratory function (3). Electrical impedance tomography (EIT) is a non-invasive, radiation-free bedside imaging technique that enables real-time monitoring of ventilation dynamics, thereby facilitating precise adjustment of mechanical ventilation settings (4). It has been suggested that EIT has the potential to mitigate lung injury in patients with respiratory distress and, when combined with esophageal manometry, can reduce the mortality rate by approximately 50% in obese patients with respiratory failure (5). In this case report, we present a rare case of severe pneumonia in an obese patient with a long history of syringomyelia who received EIT guidance to assist with ventilator weaning under conventional anti-infective therapy.

### Case presentation

**Figure 1:**
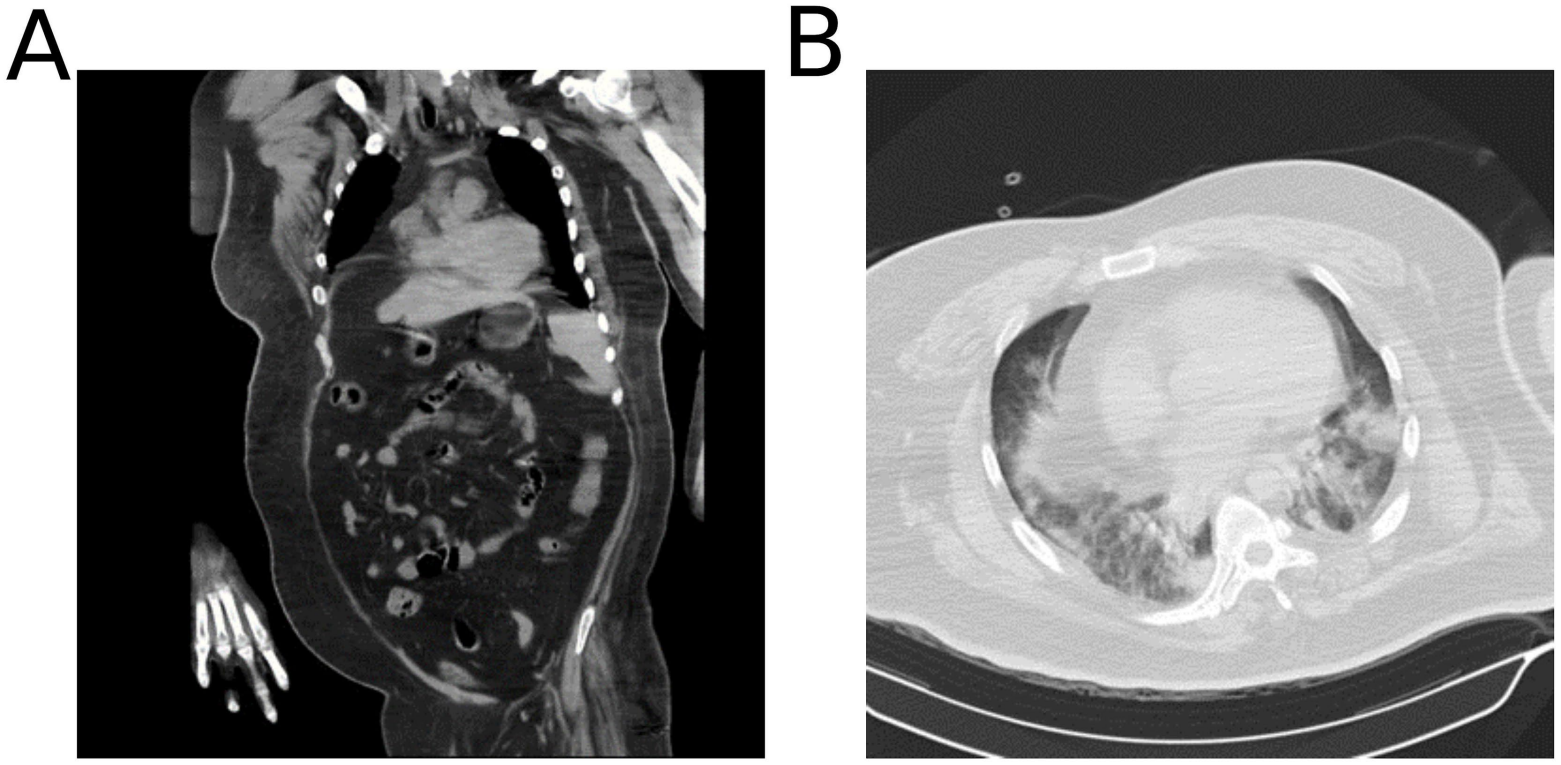
Chest radiography revealed pneumonia. **(A)** Chest radiograph and **(B)** computed tomography.

A 23-year-old man with a body mass index (BMI) of 42.9 kg/m^2^ was transferred to Zhongshan People’s Hospital in November 2023 due to progressive dyspnea of 1 week with cough and fever. The patient had self-administered cold medication for an unprovoked cough and phlegm 1 week before admission, followed by the onset of fever 3 days before admission. The patient also had a 20+ year history of syringomyelia. On admission, the patient was conscious and sedated, with pupils having a diameter of approximately 3 mm and sensitive to light reflexes, coarse lung sounds, minimal wet rales, regular cardiac rhythm, absent abdominal tenderness, diminished bowel sounds, non-edematous lower limbs, and intermittent tremors. Vital signs revealed tachycardia (137/min), hypertension (166/76 mmHg), tachypnea (42/min), hyperthermia (40.2°C), and maintained oxygen saturation at 95% under mechanical ventilation. Next-generation sequencing (NGS) results of bronchoalveolar lavage fluid suggested *Acinetobacter baumannii*, *Morganella,* and *influenza B* virus infection. There were multiple abnormalities in the patient’s blood gas analysis and laboratory test indicators at the time of admission (Supplementary Table S1). A chest computed tomography (CT) showed severe pneumonia mainly in the inferior lung lobes (Figure 1). He was then diagnosed with severe pneumonia.

**Figure 2:**
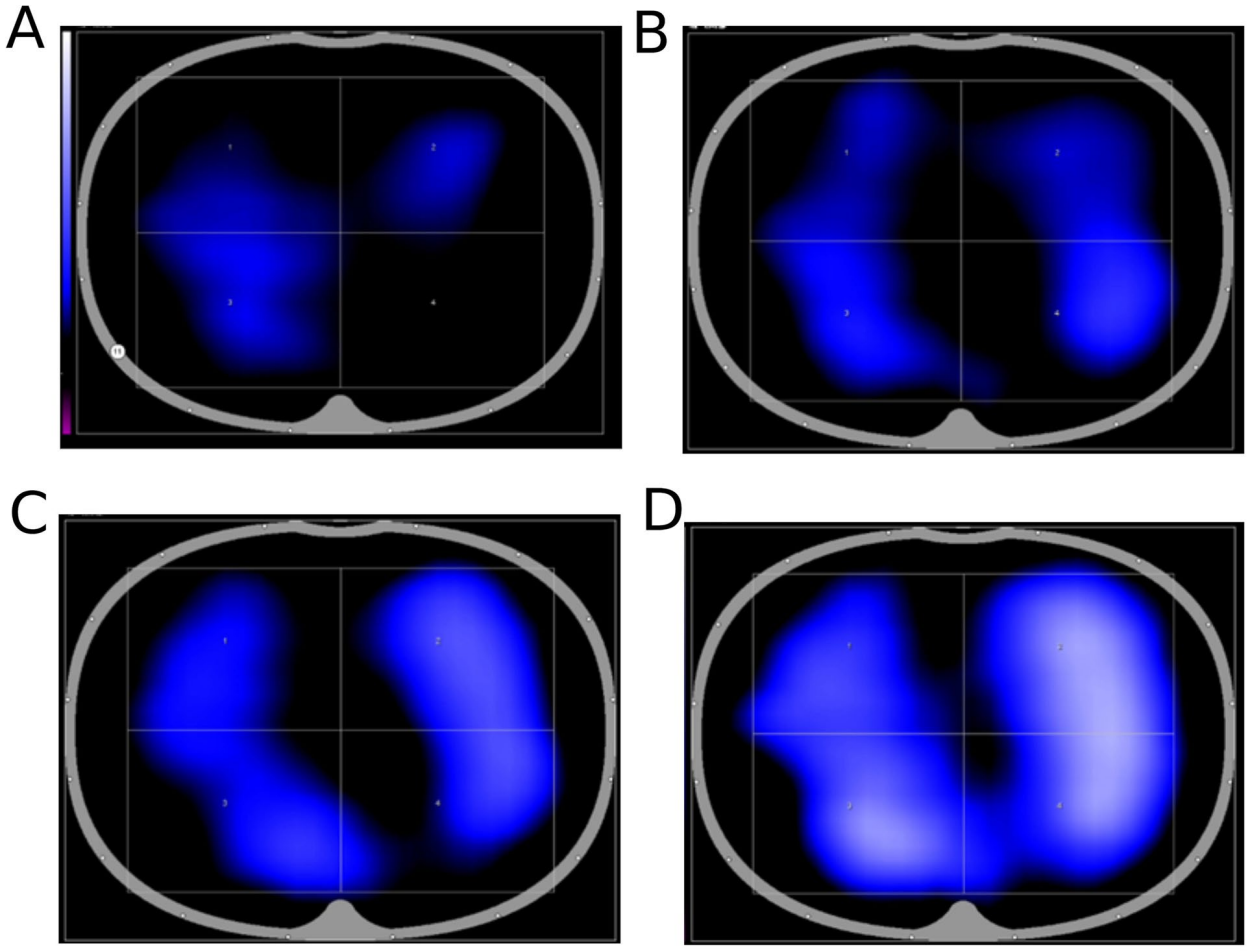
EIT measurements show ventilation in the dorsal area. **(A)** EIT assessment of ventilation function in the 45° lateral decubitus position; **(B)** EIT assessment of ventilation function in the prone position on day 3; **(C)** EIT assessment of ventilation function in the prone position on day 7; and **(D)** EIT assessment of ventilation function in successful weaning on day 28.

**Figure 3:**
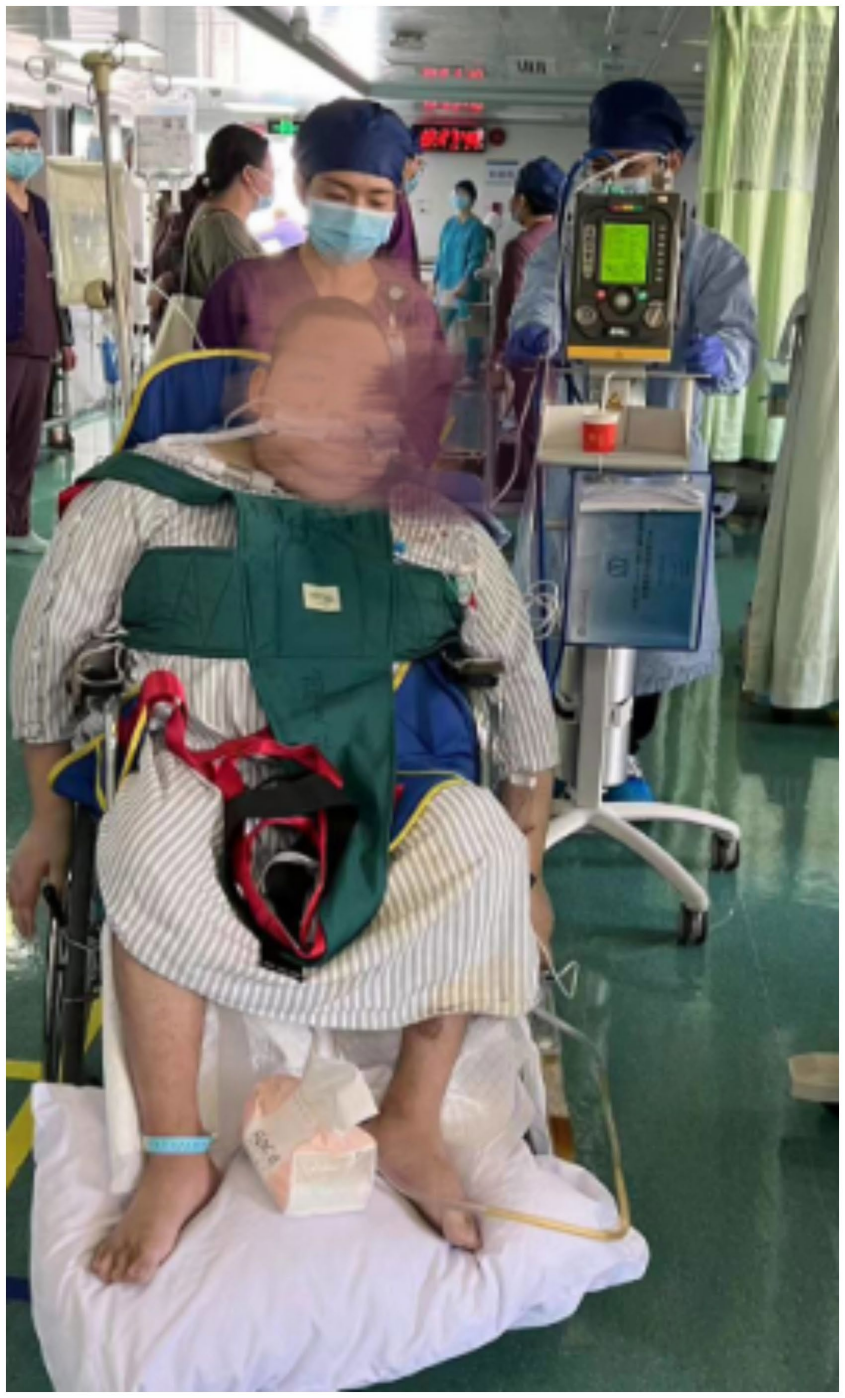
Photograph of the patient in a wheelchair during early mobilization.

The patient subsequently underwent routine lung protective ventilation and anti-infection therapy. In addition, EIT (PulmoVista 500 system by Dräger Medical, Lübeck, Germany) was conducted to guide the position. The measurement revealed suboptimal ventilation in the dorsal regions (Figure 2A). On day 3, a chest X-ray showed increased bilateral lung exudation and consolidation in the lower lobes. As the oxygenation further declined, we utilized a prone position with the head up and feet down at 30° for 16 h daily as EIT showed the most even ventilation distribution across all four regions in this configuration (Figure 2B). Starting on day 4, the parameters of the ventilator were gradually lowered to prepare for weaning. On day 7, optimal ventilation in an upright position according to EIT (Figure 2C) prompted sitting respiratory training. On day 8, the prone position was discontinued as EIT showed nearly normalized ventilation distribution. On day 9, the patient had weaning attempts that failed with obvious shortness of breath. Starting on day 11, the patient had recurrent fevers, which made weaning more difficult at this time, and began to use EIT to assist with pulmonary rehabilitation training. On day 26, the patient performed a successful SBT when 5 cm H2O PEEP during SBTs and was extubated to 5 cm H2O mask CPAP. The tracheal intubation was safely removed, and subsequently, the patient transitioned to nasal high-flow oxygen therapy. On day 28, EIT showed increased ventilation with uniform distribution (Figure 2D). With the help of an electric lift-off shift weighing machine, the patient independently completed the transfer from the bedside to the wheelchair (Figure 3). In addition, the respiratory parameters gradually normalized from day 2 to day 31 (Table 1). Meropenem was continued for anti-infection treatment, along with expectoration assistance, nutritional support, and rehabilitation exercises. On day 39, he was discharged from the hospital.

**Table 1:** Respiratory parameters of the patient during ICU admission.

Characteristics	Day 2	Day 3	Day 4	Day 9	Day 11	Day 14	Day 18	Day 22	Day 26	Day 31
Ventilatory mode	PC	PC	CPAP	CPAP	CPAP	CPAP	CPAP	CPAP	HFNC	HFNC
Tidal volume (ml/kg predicted body weight)	3.5	4	4	5	5.5	5	5	6	-	-
Respiratory rate (breaths per minute)	24	25	25	26	24	23	22	22	20	20
pH	7.35	7.42	7.39	7.45	7.42	7.45	7.42	7.44	7.40	7.44
PaCO_2_ (mmHg)	54.3	45.5	44	39	38.8	39.2	36.9	42.2	48.7	41.6
PaO_2_ (mmHg)	137	84	167.4	76	134	69	107.6	40	139.6	90.3
PEEP (cmH_2_O)	8	12	12	5	8	7	10	5	-	-
FiO_2_ (%)	60	60	60	50	60	60	70	100	50	50

PaCO_2_, arterial partial pressure of carbon dioxide; PaO_2_, denotes arterial partial pressure of oxygen; PEEP, positive end-expiratory pressure; Day 2, the day 2 of ICU admission; PC, pressure control; CPAP, continuous positive airway pressure; HFNC, high-flow nasal cannula oxygen therapy.

## Discussion

This case highlights the role of EIT in guiding the routine treatment of obese patients with severe pneumonia to achieve ventilator weaning. Guided by EIT, we meticulously optimized the patient’s ventilation position and actively facilitated early mobilization. At the outset, EIT measurements found poor ventilation in dependent regions, especially in the lower right lobe in the lateral decubitus position and in the dorsal regions in the supine position.

Therefore, we started in the early prone position and enhanced vibratory stimulation of the lower right lung to facilitate the expectoration of phlegm until it alleviated inhomogeneous ventilation distribution and improved dependent region ventilation. Since the dependent regions’ ventilation had improved, especially the lower right region, the percentage of ventilation increased from 1 to 18% on day 7, and the prone position was terminated on day 8. After the failure of weaning on day 9, considering the continuous mechanical ventilation of the patient after admission and the reduction of respiratory muscle work, along with the resulting atrophy, we intensified pulmonary rehabilitation training, including abdominal breathing, diaphragmatic breathing, vibration expectoration, and upper limb resistance training. In the orthostatic position, the EIT-monitored ventilation area was the largest and the ventilation distribution was the most homogenous. Thus, the patient received early mobilization, starting with sitting up in bed and gradually progressing to sitting at the bedside and in a wheelchair. Combined with anti-infective therapy, the obese patient underwent a successful extubation procedure and made a full recovery.

The present case report aligns with existing medical literature that has highlighted the challenges faced by obese patients with pneumonia during mechanical ventilation and the benefits of using advanced technologies to optimize their care. EIT can dynamically evaluate the changes in regional pulmonary ventilation in patients with mechanical ventilation during weaning, which is helpful in predicting the outcome of weaning. Kacmarek et al. have indicated that EIT is an essential tool to systematically assess respiratory system mechanics, to safely adjust relatively high levels of positive end-expiratory pressure (PEEP), and to improve the chances of successful weaning of obese patients (6). When compared with the use of a decremental best compliance PEEP and esophageal manometry, EIT has been shown to result in the same PEEP setting when performed in a sequential manner (7). Rubin et al. have demonstrated that incorporating EIT into a multimodal approach to individualizing ventilator management can improve outcomes, particularly in obese patients (8). EIT found that early activity and physical therapy in severe patients could improve dorsal ventilation, promote lung re-expansion, and slowly increase end-respiratory lung volume (9).

The primary strength of EIT lies in its real-time, non-invasive, and radiation-free capability to assess regional ventilation distribution, allowing for precise and individualized positioning strategies to optimize oxygenation and facilitate weaning from mechanical ventilation (10). In this case, EIT guided the adoption of prone positioning with a 30° head-up tilt, significantly improving ventilation distribution and facilitating the patient’s successful weaning from the ventilator on day 26. This tailored approach addresses the patient’s unique physiological challenges associated with severe pneumonia, emphasizing the clinical value of EIT in enhancing ventilator management. In addition, an early activity program under the guidance of EIT is also essential to the patient’s recovery. The implementation of early mobilization and rehabilitation programs in critically ill patients has been shown to reduce ICU-acquired weakness, improve functional outcomes, and potentially shorten the duration of mechanical ventilation (11).

## Conclusion

In conclusion, this study suggests that EIT may improve ventilation outcomes in obese patients with pneumonia through tailored positioning, and early mobilization and rehabilitation based on EIT findings may accelerate recovery. EIT in the care plan facilitates ventilation management in obese patients who underwent mechanical ventilation.

## Data Availability

The original contributions presented in the study are included in the article/Supplementary material, further inquiries can be directed to the corresponding author.
