# Supplementary material for: Case report: Electrical impedance tomography-guided ventilator weaning in an obese patient with severe pneumonia
Source: Front Med (Lausanne). 2025 Jan 6;11:1505114. doi: 10.3389/fmed.2024.1505114 (PMC11743170; doi:10.3389/fmed.2024.1505114)
Supplement: Supplementary file 1 [file Table_1.docx]

**Supplementary Table 1.** Blood gas and laboratory measurements of the patient at admission.

| Characteristics | Results | References |
| --- | --- | --- |
| pH | 7.37 | 7.35-7.45 |
| PaCO_2_ (mmHg) | 50.2 | 35-45 |
| PaO_2_ (mmHg) | 65.9 | 95-100 |
| PCT (ng/ml) | 4.38 | 0-0.050 |
| IL-6 (pg/ml) | 12 | 0-7.00 |
| CK (U/L) | 23328 | 41-186 |
| CK-MB (U/L) | 214 | 0-24 |
| AST (U/L) | 416 | 15-40 |
| ALT (U/L) | 63 | 9-50 |

PaCO_2_, arterial partial pressure of carbon dioxide; PaO_2_, arterial partial pressure of oxygen; PCT, Procalcitonin; IL, interleukin; CK, creatine kinase; AST, aspartate aminotransferase; ALT, alanine aminotransferase
